# Clinical characteristics and management of autoimmune enteropathy in children: case reports and literature review

**DOI:** 10.1186/s12887-023-04435-x

**Published:** 2023-11-28

**Authors:** Meng Jin, Youzhe Gong, Wenwen Liu, Xuemei Zhong

**Affiliations:** Gastroenterology Department, Children’s Hospital Capital Institute of Pediatrics, Beijing, 100020 China

**Keywords:** Infants, Autoimmune disease, Villus atrophy, Immunotherapy

## Abstract

**Background:**

Autoimmune enteropathy (AIE) defined by intractable diarrhoea and nonceliac enteropathy with villous atrophy, is a rare digestive disease. Case reports of this disease are sporadic and the clinical characteristics of AIE is seldom discussed.

**Purpose:**

We evaluate the clinical, laboratory, histopathological features, response to therapy and outcome of AIE in children.

**Method:**

We conducted a retrospective analysis of five children with AIE in our hospital. A comprehensive search of MEDLINE was performed using PubMed, through keywords of “autoimmune enteropathy, pediatric or children”. The clinical manifestations, endoscopic results, pathological results, and medication therapy of these children were collected and the cases were divided into two groups, infants (≤ 1 year old) and children (> 1 year old).

**Results:**

Five cases treated in our department: one case took eight years to make the final diagnosis; one case was positive for anti-intestinal epithelial cell (AE) antibody; three cases showed crypt apoptosis in histopathology; and two cases showed celiac-like changes. All cases were responsive to glucocorticoid therapy in the early stage of treatment, while three cases required immunosuppressant maintenance. After reviewing the literature, we performed a statistical analysis of 50 cases with a male-to-female ratio of 31:19. Among them, 35 patients (70%) were within 1 year of age, and their clinical manifestations were mainly watery stool (43 cases, 86%), weight loss (28 cases, 56%), abdominal distension (3 cases, 6%), serum AE or anti-goblet cell (AG) antibody positivity (32 cases, 64%), other immune-related antibodies (21 cases, 42%), gene mutations (9 cases, 18%), and family history (21 cases, 42%). All the children showed different degrees of intestinal villous atrophy. Thirty-seven (74%) of the children were treated early, and their clinical symptoms were relieved. Comparing the cases between different age groups, it was found that the mortality rate of children with onset in infancy was higher (P < 0.05), and there was no difference in other autoimmune diseases, AE antibody positivity rates, and other antibodies between the two groups. In addition to survival rate between different age group (P = 0. 005), there was no difference in sex, autoantibody positivity rate, single gene mutation, or family history between the two groups (P > 0.05) through analysis of mortality and clinical remission cases.

**Conclusion:**

Endoscopic examination and mucosal pathological examination should be performed to diagnose AIE in children with watery stool and weight loss who fail to be treated with diet therapy. Immunotherapy is the core of medical management of AIE and can improve prognosis. Children with a poor prognosis in infancy should be actively treated to reduce mortality rates associated with AIE.

## Introduction

Autoimmune enteropathy (AIE) is a rare intestinal disease characterised by intractable diarrhoea and immune-mediated intestinal mucosal damage [1]. The incidence of AIE is 0.06 out of 100,000 children; most cases are associated with growth retardation while some children have other comorbid autoimmune diseases [2]. Clinical manifestations of AIE include intractable diarrhoea, severe nutrient malabsorption, and hypoproteinaemia. Small intestinal villus atrophy is a main histopathological feature, among other manifestations such as lymphocytic infiltration in the lamina propria of mucosa, chronic inflammation characterized by increased or active apoptotic bodies in the crypt epithelium, and neutrophilic cryptitis. Anti-intestinal epithelial (AE) or anti-goblet cell (AG) antibodies are present in the serum. In 1978, McCarth et al. [3] reported a case of severe intestinal mucosal atrophy in a child and then proposed the concept of AIE. In 1997, based on the clinical data of 14 patients, the Mayo Clinic proposed the diagnostic criteria that are still in use today [4]: (1) chronic diarrhoea (duration > 6 weeks); (2) manifestations of malabsorption syndrome; (3) manifestations of specific small bowel lesions: partial/complete villous atrophy, deep crypt lymphocytosis, increased crypt apoptosis, and increased intraepithelial lymphocytes; (4) exclusion of other causes of villous atrophy, such as Crohn’s disease, sprue, and small intestinal lymphoma; and (5) positivity for AE and/or AG antibodies. Among them, conditions (1)–(4) are necessary for the diagnosis of AlE. Positive AE or AG antibodies can support the diagnosis; however, an absence of positive antibodies cannot exclude the diagnosis of AIE.

Diagnosis of AIE is challenging, requires small bowel biopsy, and often confused with other caoditions causing glycogenic diarrhea, celiac disease, inflammation bowel disease, gene mutations related immune deficiency disease, and so on.

Given the rare nature of AIE, most articles on this disease have been isolated case reports or case series, lacking multi-centre statistical data, especially the clinical characteristics of children at different ages. Therefore, this article will further deepen our understanding of the disease by comparing the clinical characteristics of children of different ages at time of disease onset.

## Methods

Detailed clinical data of five children diagnosed with AIE in our hospital from January 2020 to August 2022 were collected. Articles from 1982 to December 2021 were searched on Pubmed with the keyword “autoimmune enteropathy, Paediatric or children”, and the case data of all children diagnosed with AIE were obtained. A total of 69 cases involving children have been reported [5–17]. Except for 24 cases with unknown auxiliary examination data and medication therapy data, a total of 50 children were included as part of the research sample of this paper. According to the age of onset, they were divided into an infant group (≤ 1 year) and a child group (> 1 year). SPSS software was used for statistical analysis and Fisher’s exact test was used for comparison between the two groups. This study was reviewed by the Ethics Committee of the hospital (Ethics Review No: SHERLL2020026), and the guardians of five cases treated in our department signed the informed consent form.

## Patient

### Case 1

**Fig. 1:**
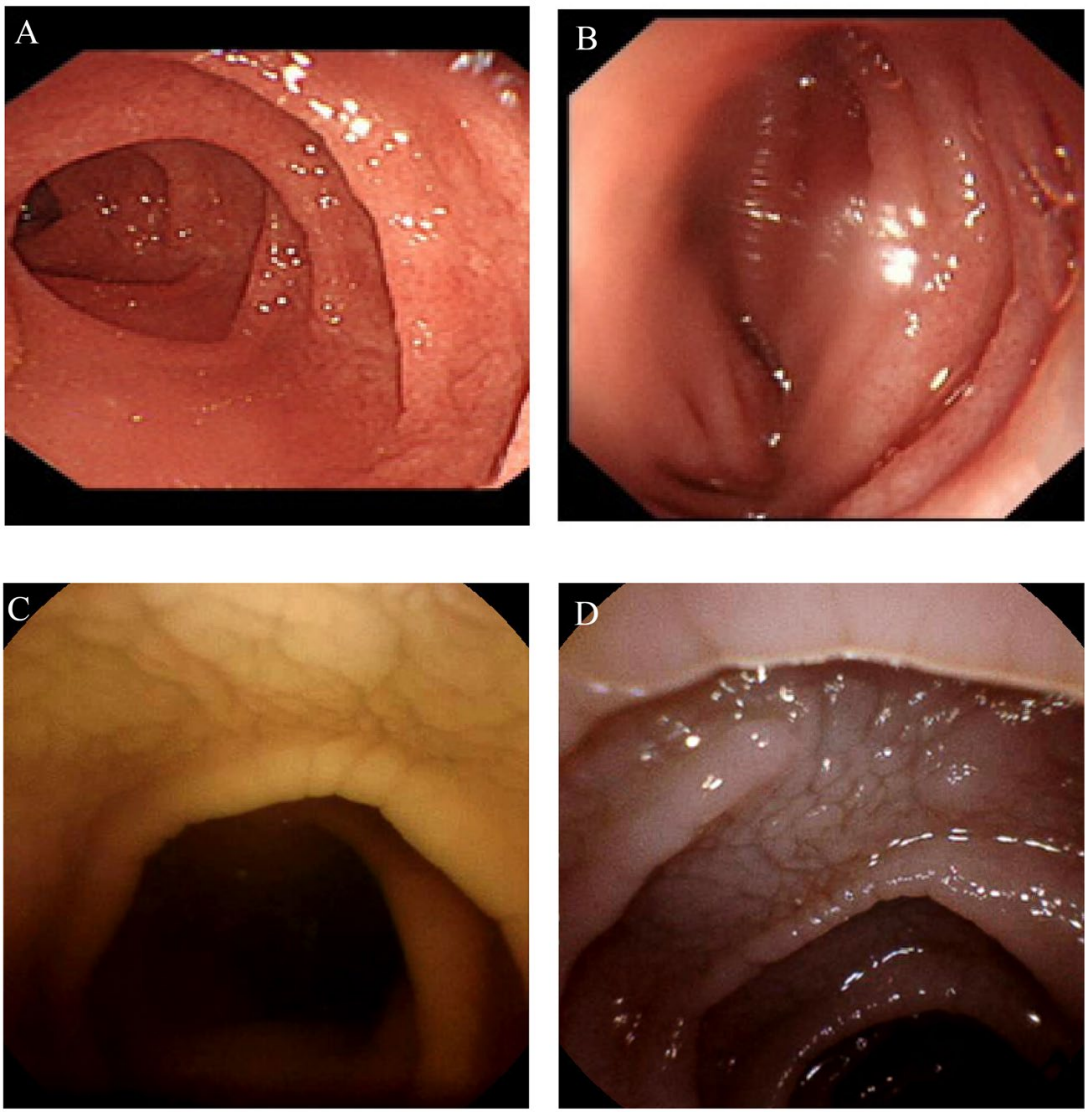
Endoscopy figures of case 1 and 4. (**A**) Showed the villi in the descending part of duodenum are low and flat. (**B**) Showed the villi of the terminal ileum are sparse, and no ulcer or erosion is found. (**C**) and (**D**) showed the small intestinal villi are flat, with celiac-like changes

**Fig. 2:**
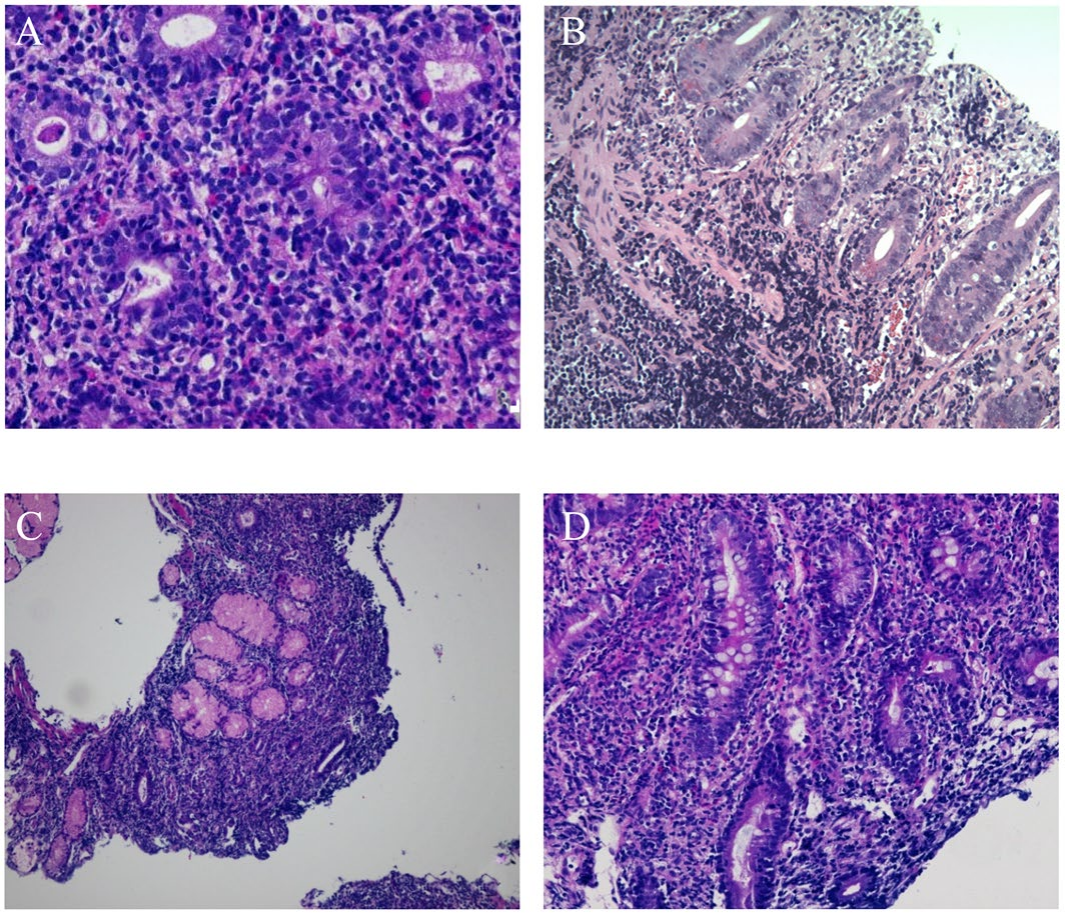
Pathological figures of case 1, 3 and 4. (**A**) HE 400 times, (**B**) HE 100 times. Shows that the villi of duodenal bulb, descending part and terminal ileum mucosa are flat or disappear, some crypts are damaged, goblet cells are significantly reduced, apoptotic cells are occasionally seen at the bottom of the crypt, and a large number of lymphocytes infiltrate into the lamina propria. Pathology showed celiac-like changes (**C**) HE 400 times, (**D**) HE 100 times The mucosal villus is severely atrophic, the lamina propria can be seen with more lymphoplasma cells and a small amount of neutrophils infiltration, crypt hyperplasia, no apoptosis, and increased intraepithelial lymphocytes

A 2 -year-old girl developed gastroenteritis after an insanitary diet at the beginning of the disease and then developed chronic diarrhoea. The clinical manifestations were watery stools and abdominal distension, which could not be relieved by diet therapy, antibiotic application, or other treatments. The stool was yellow green watery with occasional mucus and no bloody substances. During repeated monitoring, no red or white blood cells were found in the routine examination of the stool. Physical examination on admission revealed severe malnutrition. Laboratory examination:serum AE antibody was positive, decreased serum albumin. Endoscopic examination showed that the duodenal villi were low and flat (see Fig. 1A, B). The histology suggested that the small intestinal villi were atrophied (see Fig. 2A, B), apoptotic bodies were present.

### Case 2

A 5-month-old girl presented with increasing frequency of defaecation along with slow weight gain. Feeding the patient a deeply hydrolyzed formula powder was not effective. Colonoscopy showed terminal ileal oedema, a pathology suggestive of active inflammation, and the symptoms were slightly relieved by dexamethasone(1 mg/kg/day)(Due to early onset and ineffective to conventional treatment, VEO-IBD was considered by local hospital, so dexamethasone was added for treatment.). By the age of 9 months, after respiratory tract infection, the child had a large amount of watery stool and progressive weight decline. Physical examination at admission revealed severe malnutrition and a dehydrated appearance. Gastroscopy showed duodenal mucosal oedema, and pathological examination showed obvious atrophy of the duodenal mucosa, lymphocyte infiltration in the lamina propria, goblet cells, and crypt apoptosis.

### Case 3

A 9 -month-old-boy, had intermittent watery stool with slow weight gain 2 months after birth. Repeated antidiarrheal and antibiotic treatments were not effective. No ulcer or erosion was found via gastroscopy or colonoscopy in the other hospitals. Physical examination on admission revealed severe malnutrition and mild dehydration. antibody and gene detection of celiac disease were negative. Gastroscopy showed low duodenal mucosa. Pathological examination showed celiac-like changes with few neutrophil infiltrations (see Fig. 2C). Diet therapy was ineffective in this case.

### Case 4

A 10-year-old-girl had an increased defaecation frequency, 2–5 times per day, by the time she was 2 years old and slow weight gain with abdominal distension. Serum α-1 antitrypsin level was at the lower limit of normal examined by local hospital. Gastroscopy and capsule endoscopy showed chapped fissure-like changes(see Fig. 1C, D), the celiac antibody gene was negative, and enteral nutrition and single-element diet were ineffective. Physical examination at admission revealed severe malnutrition, abdominal distension, and obvious hypoproteinaemia. Gastroscopy showed celiac-like changes in the descending duodenum, and the small intestinal villi were flat, with celiac-like changes and few neutrophil infiltrations (see Fig. 2D). Diet therapy was ineffective in this case.

### Case 5

A 4-year-old girl had chronic diarrhoea, watery stool with obvious weight loss after gastrointestinal infection, and ineffective routine fasting and rehydration treatment at the beginning of the disease. Physical examination at admission revealed mild malnutrition and abdominal dilatation, while gastroscopy showed oedema in the duodenal bulb and descending part, along with sparse villi. Pathological examination showed duodenal mucosa atrophy, obvious lymphocyte infiltration in the lamina propria, and goblet cells with crypt apoptosis.

## Results

Five children in our hospital received routine rehydration therapy, parenteral nutrition support, and diet restriction after admission; but diarrhoea symptoms were not relieved. Through further examinations to exclude inflammatory bowel disease, celiac disease, stomatitis diarrhoea, small intestinal lymphoma and other diseases that may cause atrophy of small intestinal villi, a whole exon gene examination was performed, and no mutation of pathogenic site was found, which met Mayo diagnostic criteria [4], and the final diagnosis was AIE. Glucocorticoid therapy was administered at an initial dose of 2 mg/kg/day of methylprednisolone. Except for cases 4 and 5, the diarrhoea symptoms were relieved, whereas in other children, it was not immediately beneficial. After the dosage increased to 4 mg in cases 1–3, the symptoms of loose stool in cases 2 and 3 were relieved. These symptoms reappeared in the process of glucocorticoid reduction to 4 mg/day but were relieved once tacrolimus was added to the treatment course. In case 1, the symptoms were relieved by treatment with glucocorticoid combined with cyclosporine, but the symptoms reappeared in the process of adding normal diet, and rescue treatment with infliximab was ineffective. After adjusting the immunosuppressant dose to include tacrolimus, clinical remission was achieved.

Clinical characteristics of five children in our hospital are shown in Tables 1 and 2, and 3. Of the five cases, two cases had onset within 1 year of age, the mean onset age was 1.59 years old and the average time from onset to final diagnosis was 2.4 years. All children had malnutrition, anaemia, and hypoproteinaemia and were responsive to glucocorticoid therapy. One case in which the drug was completely stopped, and the other three cases needed immune drugs to be maintained, there is also one patient with hormone reduction under observation.

Statistical analysis was performed on the clinical data of the 50 children (Table 4). The ratio of males to females was 31:19, with boys being more affected by AIE. The average age of onset was 1.6 years old, while 35 cases (70%) experienced onset within 1 year of age, among them 27 were onset within 6 months, and the average age of whom was 2.5 months. Clinical manifestations were mainly watery stools (43 cases, 86%), weight loss (28 cases, 56%), abdominal distension (3 cases, 6%), and positive erum AE or anti-goblet cell (AG) antibody (32 cases, 64%). Other immune-related antibodies were detected in 21 cases (42%): anti-thyroid antibody in 6 cases; anti-nuclear antibody in 10 cases; anti-insulin antibody in 2 cases; anti-Ergot antibody in 1 case; and anti-Saccharomyces cerevisiae antibody in 2 cases. Twenty-one children (42%) had combined family histories of AIE. All children had intestinal mucosal pathology showing different degrees of atrophy.

Gene mutations related disease were found in a total of 9 cases (18%): two cases immunodysregulation polyendocrinopathy enteropathy X-linked (IPEX),three cases hypogammaglobulinemia, one case autoimmune polyendrocrinopathy-cadidiasis-ectodermal dystrophy (APECED), one case Kabuki syndrome, one case X-linked immunodeficiency, and one case hyper-IgE syndrome.

42 cases received drug treatment after diagnosis (Table 5), 7 cases of simple glucocorticoid, 18 cases of tacrolimus, 9 cases of azathioprine, 2 cases of cyclosporine, 2 cases of cyclophosphamide, and 4 cases of maintenance therapy with parenteral nutrition. Thirty-seven cases achieved clinical remission after drug treatment (74%), five cases died of infection after adding immunosuppressants, and eight cases died without medication.

The children in our study were divided into infant (≤ 1 year old) and child (> 1 year old) groups. The chi-square test was used for the analysis (Table 6). The mortality rate of children with onset in infancy was higher (P = 0.005, < 0.05). There was no significant difference in other autoimmune diseases, in the AE antibody-positive rate, and other antibodies between the two groups. In addition to age group, there was no difference in sex, autoantibody positivity rate, single gene mutation, or family history between the two groups through analysis of death and clinical remission case groups (Table 7).

**Table 1:** **Clinical characteristics of 5 AIE patients of our hospital**

No.	Sex	Onset age(Y)	Time for diagnosis(m)	Clinical manifestations	Z-score weight for height	Family history
1	F	2.2	4	Watery stools, abdominal distension	-3.5	N
2	F	0.4	6	Watery stools	-1.7	N
3	M	0.16	7	Watery stools	-5.1	N
4	F	2	120	Mushy stool, abdominal distension	-3.15	N
5	M	3.2	9	Watery stools, abdominal distension	0.3	N

F: Female, M: Male, Y: Year, m: Month, N: Negative

**Table 2:** **Laboratory examinations of 5 AIE children of our hospital**

No.	Hb	Alb	AE-IgG	Celiac antibody	Electronic gastroscope	Electronic colonoscope	Capsule endoscope	Pathogenic detection	Other antibodies	Gene testing
1	85	25	P	N	Duodenal villi low and flat	Edema of terminal ileum mucosa	Not done	N	N	N
2	78	29	N	N	Duodenal mucosal oedema	Sparse villi in terminal ileum mucosa	Not done	fungal infection, cure	N	N
3	68	25	N	N	Low duodenal mucosa	N	Not done	N	Anti-thyroid antibody	N
4	98	27	N	N	Celiac-like changes in the descending duodenum	N	Lower and flat villi of upper small intestine	N	Anti-Saccharomyces cerevisiae antibody	N
5	102	26	N	N	Oedema in the duodenal bulb and descending part, along with sparse villi	N	The villi of the upper jejunum are low and flat	N	Anti-Saccharomyces cerevisiae antibody	N

Hb: Hemoglobin, g/L, Alb: Albumin, g/L, AE-IgG: Anti-intestinal epithelial immunoglobulin G, P: Positive, N: Negative

**Table 3:** **Endoscopy, treatment and outcome of 5 AIE children of our hospital**

No.	Endoscopy	Pathological type	Treatment	Outcome	Follow up
1	Villi low and flat	Duodenitis type	Glucocorticoid -Cyclosporin-Remicade-Tacrolimus	No diarrhea, weight gain	Tacrolimus maintenance
2	Villi low and flat	Duodenitis type	Glucocorticoid	Normal defecation and growth	No repetition after drug withdrawal
3	Villi low and flat	Celiac-like	Glucocorticoid - Tacrolimus	Defecate 1–2 times per day	Tacrolimus maintenance
4	Villi low and flat	Celiac-like	Glucocorticoid	Defecate 1 times per day, weight gain	Glucocorticoid reduction
5	Villi low and flat	Duodenitis type	Glucocorticoid - Tacrolimus	Defecate 1 times per day, normal weight	Tacrolimus maintenance

**Table 4:** **The clinical features of 50 AIE children**

Clinical features	N = 50
**Sex**	
M	31(62%)
F	19(38%)
Age(Y)	Average: 1.6y
≤ 1Y	35(70%) Average: 4.2 m
<6 m	27(54%) Average: 2.5 m
≥6 m &≤1Y	8(16%) Average: 10 m
> 1Y	15(30%)
**Clinical manifestation (Onset symptoms)**	
Watery stools	43(86%)
Weight loss	28(56%)
Watery stools and weight loss at the same time	27(54%)
Abdominal distension	3(6%)
Vomit	1(2%)
**Laboratory examination**	
Positive AE antibody	32(64%)
Positive other antibodies	21(42%)
Gene mutations related disease	9(18%)
Mucosal atrophy	50(100%)
Positive family history	21(42%)

**Table 5:** **The clinical therapeutic and outcome of 50 AIE children**

Clinical therapeutic	N = 50	Outcome
Simple glucocorticoids	7(14%)	Clinical remission
Immune drugs	31(62%)	26 cases clinical remission(83%)
Tacrolimus	18	4 cases died, 14 cases Clinical remission(77%)
Azathioprine	9	1 case died, 8 cases Clinical remission(88%)
Cyclosporine	2	Clinical remission
Cyclophosphamide	2	Clinical remission
Parenteral nutrition	4(8%)	Partial remission of Intestinal symptoms, but no remission of growth retardation

**Table 6:** **The analysis of clinical features of the AIE patients from different ages**

	AE antibody		Other antibodies		Genetic defect		Survival rate	
Age	N	P	N	P	N	P	Survival	Death
≤ 1Y	14	21	21	14	30	5	22	13
>1Y	4	11	8	7	11	4	15	0
Chi-square	0.834		0.192		1.034		11.126	
P Value	0.523		0.662		0.423		0.005	

**Table 7:** **The analysis of survival and outcome of the AIE patients from different ages**

Survival	Sex		Family history		Genetic defect		AE antibody		Other antibodies		Family history and ≤ 1Y		Family history and >1Y	
	F	M	N	P	N	P	N	P	N	P	N	P	N	P
Relieve	16	21	19	18	31	6	24	13	20	17	11	26	7	30
Death	3	10	10	3	10	3	8	5	9	4	3	10	0	13
Chi-square	1.746		2.718		0.294		0.046		0.932		0.217		4.603	
P Value	0.32		0.191		0.679		1		0.515		0.191		0.668	

## Discussion

Autoimmune enteropathy (AIE) is an autoimmune disease of unknown aetiology, characterised by villous atrophy of the small intestinal mucosal epithelium. Most of these cause chronic diarrhea and may be life-threating diarrhea in infancy.

The main clinical manifestation of AIE is severe and long-term secretory diarrhoea, which is characterised by a large amount of watery stools, with a diarrhoea volume of up to 5,000 mL/day. The effects of fasting and water deprivation are not typically beneficial and the faecal aetiology is negative. Parenteral nutrition is required to maintain water and electrolyte balance. Intestinal malabsorption can result in low body weight and poor growth [18]. In addition, children with AIE are often prone to local and systemic infections related to the skin barrier, intestinal barrier, immunotherapy, and malnutrition [19]. In this paper, five children who were clinically relieved by immunotherapy developed systemic infections and died. Endoscopic manifestations are mostly mild, with sparse or oedema of the small intestinal villi, and some of them show fan-shaped and fissure-like changes in the duodenum, which are difficult to distinguish from celiac disease. AIE can be isolated in the gastrointestinal tract or may be part of a systemic disease. In children, these systemic diseases are mostly primary immunodeficiency diseases that are mostly related to gene mutations [5, 20]. Common diseases include immune disorders, multiple endocrine diseases, enteropathy, autologous multiple endocrine diseases, candidiasis, ectodermal dystrophy syndrome, and others [21].

Laboratory tests for AIE lack specificity, and AE and/or AG antibodies are only present in the serum intestinal autoantibodies of some children. Positive serum AE or AG antibodies increase the possibility of AIE diagnosis, but is not necessary for diagnosis [22]. It has been reported that serum intestinal autoantibodies appear only after the onset of mucosal injury and decrease or become negative with treatment before histological remission [23]. Although many experts currently recommend their use as diagnostic criteria for AIE, the retrospective analysis in this paper shows that the positive rate of antibodies is only 64%. The presence of antibodies cannot be detected in all patients with typical AIE and not in other diseases either, such as inflammatory enteropathy, celiac disease, and HIV [23–26]. In addition, peripheral blood autoantibodies that can be detected in AIE patients include anti-thyroglobulin, anti-thyroid peroxidase, and anti-Saccharomyces cerevisiae antibodies; however, their significance and diagnostic value in the pathogenesis of AIE are still unclear [27]. Our retrospective statistical analysis also concluded that there was no difference between the presence of autoantibodies and remission rate and prognosis of treatment.

AIE has no specific histological features; however, all patients have gastrointestinal mucosal injuries. Injury is usually confined to the mucosa, while deep ulcers or transmural inflammatory changes are rarely observed. The pathological diagnostic criteria of AIE in the past were blunt villi, lymphocytosis in the deep crypt, increased apoptosis of crypt cells, and increased lymphocytes in the surface intraepithelium; however, at present, the pathological tissues of AIE can be divided into four types: active duodenitis (characterised by active chronic inflammatory process and neutrophil cryptitis, but without abscess), celiac disease-like, graft-versus-host disease-like (obvious apoptosis can be seen in crypts), and mixed mode. At present, all four types have been reported in paediatric patients [5, 20, 28]. A retrospective study of the mucosal pathology of AIE showed that among 23 children, there were 10 celiac-like (43.3%), eight mixed mode, one acute GVHD mode, and four duodenitis modes, while for patients with immunodeficiency, the prevalence of celiac-like mode could be as high as 72% [6]. Two of the confirmed cases in our hospital also showed a celiac-like pattern; therefore, this pattern is still very common among children, which is important in early differentiation and diagnosis.

These results suggest that the prognosis of patients with AIE depends on the severity of symptoms and signs of the digestive system, the severity and extent of histological lesions of the gastrointestinal tract, and the existence of parenteral involvement [27, 29]. However, through retrospective analysis of case data, it was found that only the age of onset was closely related to mortality (Table 2), whereas there was no difference in family history, signs, or other diseases between the age groups. This may also be due to the fact that the number of cases we collected was mostly from the literature, and the purpose of case studies in the literature is specific and the description of pathological changes is different, which has a certain impact.

At present, there is no consensus on the treatment of AIE, because children with AIE often suffer from stunting due to nutritional absorption disorder, and their developmental conditions such as weight and height are mostly lower than those of normal children of the same age. The use of immunosuppressants based on nutritional support is recommended [21, 23]. At present, there is no evidence-based treatment scheme owing to the lack of a large-scale sample and controlled trial evaluation. Glucocorticoids are the first choice for immunosuppressive therapy; when glucocorticoids are ineffective, tacrolimus, cyclosporine, tumour necrosis factor α monoclonal antibody, and other second-line treatment schemes can be selected for immunosuppressive therapy. Tacrolimus has been reported to have a high clinical remission rate (approximately 77%) for steroid-ineffective or refractory autoimmune bowel disease [23], which is similar to the remission rate of the cases we collected (78%). All cases in this study were treated with hormone and immunosuppressive therapy, and most of them had clinical remission. A few children died of septicaemia, but the mortality rate was significantly lower than that of the untreated children. Therefore, early drug treatment can save the lives of children with AIE.

Overall, the clinical and histopathological manifestations of AIE in children, especially in infants, are quite varied. Early diagnosis and medication can reduce mortality and improve growth and development. Therefore, it is helpful for clinicians and pathologists to identify the clinical characteristics and different pathological patterns of AIE in its early stages.

This paper has limitations. Since the clinical data of 45 children were reported in the previous literature, the clinical remission time and follow-up after drug addiction were not fixed, so the overall evaluation and long-term clinical remission rate of drug treatment were uncertain. Therefore, the follow-up will strengthen the follow-up work of the confirmed patients in our hospital and help to evaluate the medication and clinical prognosis.

## Data Availability

The data have been listed above in the main paragraph.
